# Characterization of *Mycobacterium orygis* as *M. tuberculosis* Complex Subspecies

**DOI:** 10.3201/eid1804.110888

**Published:** 2012-04

**Authors:** Jakko van Ingen, Zeaur Rahim, Arnout Mulder, Martin J. Boeree, Roxane Simeone, Roland Brosch, Dick van Soolingen

**Affiliations:** Radboud University Nijmegen Medical Centre, Nijmegen, the Netherlands (J. van Ingen, M.J. Boeree, D. van Soolingen);; International Center for Diarrheal Disease Research, Bangladesh, Dhaka, Bangladesh (Z. Rahim);; National Institute for Public Health and the Environment, Bilthoven, the Netherlands (A. Mulder, D. van Soolingen);; Institut Pasteur, Paris, France (R. Simeone, R. Brosch)

**Keywords:** Tuberculosis and other mycobacteria, oryx bacillus, molecular typing, epidemiology, Mycobacterium orygis, bacteria

## Abstract

The oryx bacilli are *Mycobacterium tuberculosis* complex organisms for which phylogenetic position and host range are unsettled. We characterized 22 isolates by molecular methods and propose elevation to subspecies status as *M. orygis*. *M. orygis* is a causative agent of tuberculosis in animals and humans from Africa and South Asia.

Traditionally, the *Mycobacterium tuberculosis* complex comprises tubercle bacilli of 8 distinct subgroups: *M. tuberculosis*, *M. africanum*, *M. canettii*, *M. bovis*, *M. caprae*, *M. pinnipedii*, *M. microti*, and *M. mungi* (*1**–**4*). Two other distinct branches of the *M. tuberculosis* complex phylogenetic tree exist, the dassie and oryx bacilli, causative agents of tuberculosis in the animal species after which they are named. Neither has been validly described as separate taxa, nor have they been associated with disease in humans (*1**–**4*).

Oryx bacilli have been isolated from members of the *Bovidae* family, i.e., oryxes, gazelles (*3*), deer, antelope, and waterbucks (*5*), although their exact host range remains unsettled. No human disease caused by the oryx bacilli has been reported. These bacilli most likely constitute a separate phylogenetic lineage; however, their exact position has not been established with valid phylogenetic markers, such as large genomic deletions or single nucleotide polymorphisms (SNPs). To settle the phylogenetic position and host range of the oryx bacilli, we collected all oryx bacillus isolates from our laboratory database to establish their sources and subjected the isolates to extended phylogenetic analysis.

## The Study

**Figure 1:**
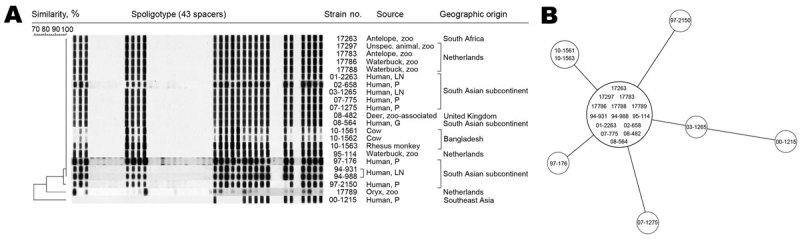
Spoligotyping and 24-locus variable number tandem repeat (VNTR) typing results for *Mycobacterium orygis*. A) Spoligotyping patterns for the oryx bacillus isolates in this study; ST587 is the most common pattern (labeled SB0422 at www.mbovis.org), with minor deviations. B) Minimum spanning tree based on 24-locus VNTR typing results for the oryx bacillus isolates in this study. One type dominates, with few strains representing minor variations. The dominant clone includes isolates from humans and animals. P, pulmonary; LN, lymph node; G, gastric juice. Both panels were created by using BioNumerics version 6.1 software (Applied Maths, Sint-Martens-Latem, Belgium); similarity coefficients were calculated by using Dice (spoligotyping) and Pearson (VNTR) methods; cluster analysis was done by the UPGMA (unweighted pair group method with arithmetic mean). Isolate 10–1562 (cow, Bangladesh) could not be included in (B) because of insufficient DNA.

We selected 22 isolates on the basis of >90% similarity of the IS*6110* restriction fragment-length polymorphism (RFLP) pattern to that of established and previously published oryx bacillus strains; 11 isolates originated from animals, and 11 originated from 10 human patients (Figure 1) (*1**–**3*). All isolates yielded smooth to greasy domed nonchromogenic colonies in culture (Technical Appendix Figure).

For phylogenetic analysis, we performed SNP and region of difference (RD) analysis (*2**,**6*). RD and SNP typing showed a consistent pattern among the isolates, with presence of regions RD1, RD2, RD4, RD5a (*Rv2348*), RD6, and RD13–RD16 and absence of regions RD3, RD5b (*plcA*), and RD7–RD12 (Technical Appendix Table 1). The deleted region for RD12 (RD12^oryx^) was larger than that for *M. bovis* and *M. caprae*. Analysis of the flanking regions indicated an IS*6110* insertion at the *M. tuberculosis* H37Rv coordinates of 3479670 and 3491252 with deletion of the intermediate area covering the open reading frames of the *Rv3111* to *Rv3125c* genes (Technical Appendix Table 2). Isolates also showed the RDoryx_1, RDoryx_4, and RDoryx_wag22 deletions and the *mmpL6*^551^AAG mutation (Technical Appendix Table 1). Results agreed with those from previous studies (*1**,**6*).

Using pncA-1F 5′-GGC CGC GAT GAC ACC TCT-3′, pncA1-R 5′-GCC GCA GCC AAT TCA GCA GT-3′, pncA-2F 5′-CGA AGC GGC GGA CTA CCA TCA CG-3′, and pncA-2R 5′-CCC CAC CTG CGG CTG CGA ACC-3′ primers, we partially sequenced *Rv2042c*, *Rv2044c*, and the full *pncA* gene. The *pncA* sequences of the isolates from animals and humans were identical to those of *M. tuberculosis* H37Rv; in codon 38 of the *Rv2042c* gene, directly upstream from *pncA*, a GTC to GGC (Ser→Ala) mutation was noted in all 22 isolates; the partial *Rv2042c* sequence is stored in GenBank (accession no. JF417976). To assess the specificity of the *Rv2042*^38^ GGC mutation, we screened 2 isolates of all *M. tuberculosis* complex (sub)species and 2 isolates of all *M. tuberculosis* groupings, on the basis of >60% IS*6110* similarity, for this mutation; we did not find it in any of the strains tested (data not shown).

We performed spoligotyping and 24-locus mycobacterial interspersed repetitive units–variable-number tandem repeat (MIRU-VNTR) typing, as described (*7**,**8*). Spoligotyping mostly showed the sequence type (ST) 587 pattern in the spolDB4 database and labeled *M. africanum* (*9*); minor variations in spoligotype were observed (Figure 1, panel A). All isolates had unique IS*6110* RFLP patterns, although with >75% similarity; patterns were characterized by high (i.e., 17–20) numbers of IS*6110* copies (data not shown). VNTR typing showed closely related patterns (Table A1). A minimum spanning tree showed the clonality of the *M. orygis* isolates (Figure 1, panel B). The GenoType MTBC assay (Hain Lifesciences, Nehren, Germany) identified all isolates as *M. africanum*.

Baseline clinical data of humans were extracted from the anonymized National Tuberculosis Register. Ethical approval was waived for this retrospective laboratory-based study. Nine of the 10 human patients were of South Asian origin; the other was of Southeast Asian origin (Figure 1); patients’ average age was 41 years (range: 0–69 years). Clinically, 6 patients had pulmonary tuberculosis, 3 had lymphadenitis, and 1 child had tuberculosis diagnosed by gastric fluid culture. All isolates were susceptible to all first-line antituberculosis drugs, including pyrazinamide, and hence the standard treatment regimen was started for all patients. Patients received treatment for an average of 9 months; no details about individual regimens were available. No bacteriologically proven relapses were noted. No information was available about contact-tracing studies.

## Conclusions

**Figure 2:**
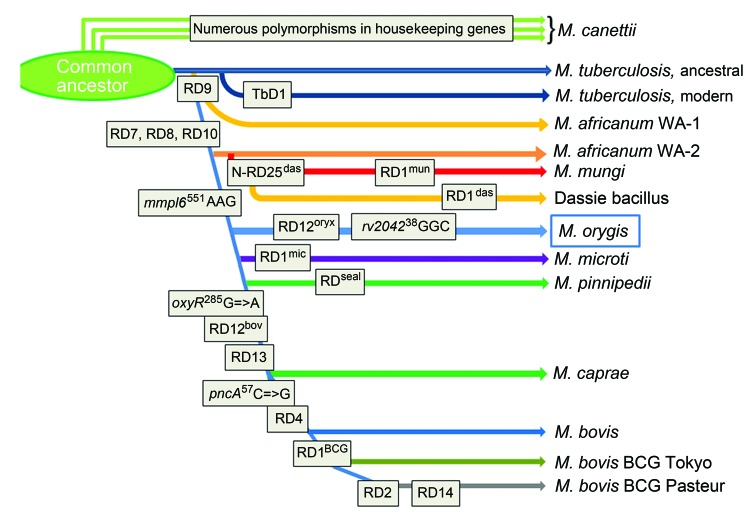
Updated phylogeny of the *Mycobacterium tuberculosis* complex based on the findings of Brosch et al. (*2*). Combined findings place *Mycobacterium orygis* at a distinct phylogenetic position between the *M. africanum*/dassie bacillus/*M. mungi* cluster and *M. microti*.

The oryx bacillus is a phylogenetically distinct lineage of the clonal *M. tuberculosis* complex and thus deserves a separate subspecies status; we propose the name *M. orygis* (Latin: oryx, genitive: *orygis*, of the oryx) to convey that this subspecies was first characterized after its isolation from an oryx (Figure 2).

The most common spoligotype (ST587) is present in the spolDB4 database and labeled *M. africanum* (*9*). The *M. orygis* bacteria share the *gyrB*^1450^ (G→T) mutation with *M. africanum*, *M. microti*, and *M. pinnipedii* (*1*). Hence, the GenoType MTBC assay identifies *M. orygis* as *M. africanum*. Thus, *M. orygis* isolates may have previously been misidentified as *M. africanum* (*9**,**10*).

The animal-adapted *M. tuberculosis* complex lineage is thought to have evolved in Africa when an *M. africanum*–like clone diverged from *M. tuberculosis*, as shown by the loss of the RD9 locus. Consecutive loss of DNA during the adaptation to novel hosts led to the distinct subspecies with its distinct host range that we know today (*1**,**4**,**5**,**11*). This matches geographically with the habitats of *Oryx* species, gazelles, and waterbucks.

For *M. orygis*, the host range remains unknown but may include oryxes, waterbucks, and gazelles in eastern Africa and the Arabian Peninsula; cows and rhesus monkeys in South Asia; and humans. The evolutionary explanation for the diversity in geographic distribution and hosts of *M. orygis* remains elusive. This diversity contrasts starkly with the conserved VNTR and spoligotype patterns.

The presence of *M. orygis* in diseased cows and a monkey in Bangladesh, unique RFLP patterns, and lack of onward transmission suggest animal-to-human transmission. As for *M. bovis*; humans may be accidental, dead-end hosts.

*M. orygis*, unlike *M. microti*, the dassie bacillus, and *M. mungi*, shows an intact RD1 region. This region encodes part of the virulence-related ESX-1 secretion system of tubercle bacilli (*12*).

Molecular characteristics define the isolates previously labeled as oryx bacilli as a distinct subspecies in the *M. tuberculosis* complex for which we propose the name *M. orygis*. The *Rv2042*^38^ GGC mutation is a novel, useful genetic marker to identify *M. orygis*, which is otherwise characterized by the presence of genomic regions RD1, RD2, RD4, RD5a (*Rv2348*), RD6, RD13–RD16, and the *mmpL6*^551^AAG polymorphism, with absence of regions RD3, RD5b (*plcA*), RD7–RD12, RDoryx_1, RDoryx_4, and RDoryx_wag22. The deletion of RD12 is subspecies specific. Isolates yield the ST587 or closely related spoligotypes, 17–20 copies of IS*6110*, and a distinct 24-locus VNTR pattern with minor variations. *M. orygis* is a causative agent of tuberculosis in oryxes, gazelles, and waterbucks of African origin; cows and rhesus monkeys of South Asian origin; and humans.

## Supplementary Material

Technical Appendix Colony morphology of *Mycobacterium orygis* on Stonebrink medium, molecular typing results in characterization of *M. orygis* as *M. tuberculosis* complex subspecies, and functional analysis of genes in the RD12 deletion area of *M. orygis*.

## Appendix

**Table A1:** Twenty-four locus variable number tandem repeat typing results in characterization of *Mycobacterium orygis* as *M. tuberculosis* complex subspecies*

Strain	Variable number tandem repeat locus																							
	580	2996	802	960	1644	3192	424	577	2165	2401	3690	4156	2163b	1955	4052	154	2531	4348	2059	2687	3007	2347	2461	3171
17263	3	4	2	7	4	4	2	5	x	4	4	3	x	3	2	2	4	2	2	1	3	3	2	3
17297	3	4	2	7	4	4	2	5	x	4	4	3	x	3	2	2	4	2	2	1	3	3	2	3
17783	3	4	2	7	4	4	2	5	x	4	4	3	x	3	2	2	4	2	2	1	3	3	2	3
17786	3	4	2	7	4	4	2	5	x	4	4	3	x	3	2	2	4	2	2	1	3	3	2	3
17788	3	4	2	7	4	4	2	5	x	4	4	3	x	3	2	2	4	2	2	1	3	3	2	3
17789	3	4	2	7	4	4	2	5	x	4	4	3	x	3	2	2	4	2	2	1	3	3	2	3
94–931	3	4	2	7	4	4	2	5	x	4	4	3	x	3	2	2	4	2	2	1	3	3	2	3
94–988	3	4	2	7	4	4	2	5	x	4	4	3	x	3	2	2	4	2	2	1	3	3	2	3
95–114	3	4	2	7	4	4	2	5	x	4	4	3	x	3	2	2	4	2	2	1	3	3	2	3
97–176	3	4	**3**	7	4	4	2	5	x	4	4	3	x	3	2	2	4	2	2	1	3	3	2	3
97–2150	3	4	2	**9**	4	4	2	**6**	x	4	4	3	x	3	2	2	4	2	2	1	3	3	2	3
00–1215	3	4	2	7	4	**3**	2	5	**3**	4	4	3	x	3	2	2	4	2	**1**	1	3	3	2	3
01–2263	3	4	2	7	4	4	2	5	x	4	4	3	x	3	2	2	4	2	2	1	3	3	2	3
02–658	3	4	2	7	4	4	2	5	x	4	4	3	x	3	2	2	4	2	2	1	3	3	2	3
03–1265	3	4	2	7	4	**3**	2	5	x	4	4	3	x	3	2	2	4	2	2	1	3	3	2	3
07–775	3	4	2	7	4	4	2	5	x	4	4	3	x	3	2	2	4	2	2	1	3	3	2	3
07–1275	3	4	2	7	4	4	2	5	x	**5**	4	3	x	3	2	2	4	2	**1**	1	3	3	2	3
08–482	3	4	2	7	4	4	2	5	x	4	4	3	x	3	2	2	4	2	2	1	3	3	2	3
08–564	3	4	2	7	4	4	2	5	x	4	4	3	x	3	2	2	4	2	2	1	3	3	2	3
10–1561	3	4	2	**6**	4	4	2	5	x	4	4	3	x	3	2	2	4	2	2	1	3	3	2	3
10–1563	3	4	2	**6**	4	4	2	5	x	4	4	3	x	3	2	2	4	2	2	1	3	3	2	3

*x, unamplifiable. **Boldface** indicates deviations from the most frequently observed number of repeats.
