# Supplementary material for: Characterization of Mycobacterium orygis as M. tuberculosis Complex Subspecies
Source: Emerg Infect Dis. 2012 Apr;18(4):653–5. doi: 10.3201/eid1804.110888 (PMC3309669; doi:10.3201/eid1804.110888)
Supplement: Technical Appendix — Colony morphology of Mycobacterium orygis on Stonebrink medium, molecular typing results in characterization of M. orygis as M. tuberculosis complex subspecies, and functional analysis of genes in the RD12 deletion area of M. orygis. [file 11-0888-Techapp_2p.pdf]

Article DOI: http://dx.doi.org/10.3201/eid1804.110888

Characterization of *Mycobacterium orygis* as *M. tuberculosis* Complex Subspecies

Technical Appendix

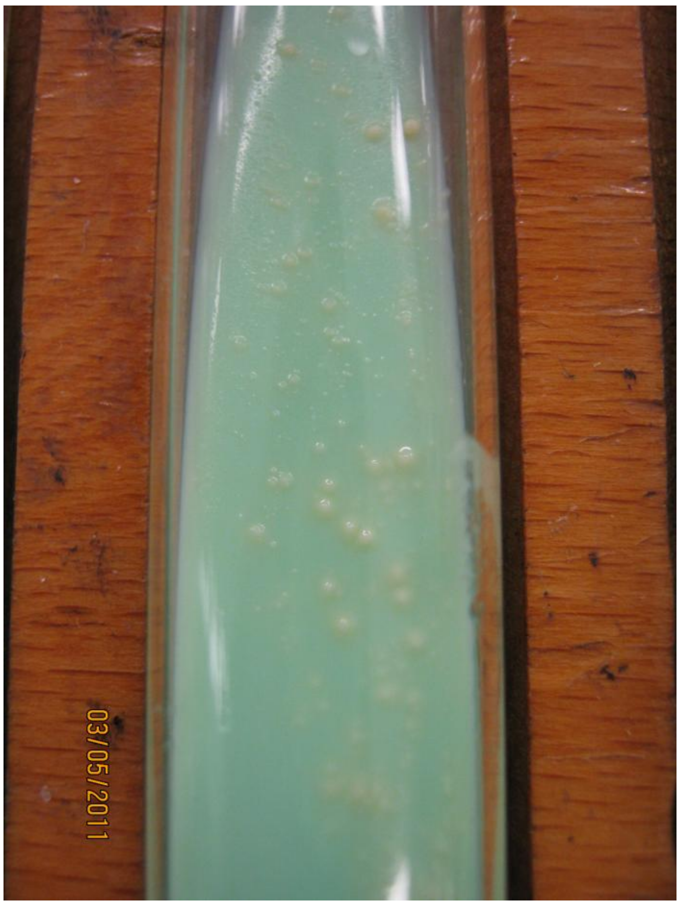

Technical Appendix Figure. Colony morphology of *Mycobacterium orygis* on Stonebrink medium.

Technical Appendix Table 1. Molecular typing results in characterization of *Mycobacterium orygis* as *M. tuberculosis* complex subspecies*

Strain no.	Origin of sample	mmpL6 codon 551	RD1	RD2	RD3	RD4	RD5a Rv2348	RD5b plcA	RD6	RD7	RD8	RD9	RD10	RD11	RD12† Oryx	RD13	RD14	RD15	RD16	RDory x1	RDory x4	wag22
01-2263	Human, LN	AAG	1	1	0	1	1	0	1	0	0	0	0	0	0	1	1	1	1	0	ND	ND
97-2150†	Human, P	AAG	1	1	0	1	1	0	1	0	0	0	0	0	0	1	1	1	1	0	0	0
00-1215†	Human, P	AAG	1	1	0	1	1	0	1	0	0	0	0	0	0	1	1	1	1	0	ND	0
97-176†	Human, P	AAG	1	1	0	1	0	0	1	0	0	0	0	0	0	1	1	1	1	0	ND	0
07-1275	Human, P	AAG	1	1	0	1	1	0	1	0	0	0	0	0	0	1	1	1	1	0	0	0
07-775	Human, P	AAG	1	1	0	1	1	0	1	0	0	0	0	0	0	1	1	1	1	0	0	0
03-1265	Human, LN	AAG	1	1	0	1	1	0	1	0	0	0	0	0	0	1	1	1	1	0	0	0
94-988† &94-931†	Human, LN	AAG	1	1	0	1	1	0	1	0	0	0	0	0	0	1	1	1	1	0	ND	0
08-564	human, G	AAG	1	1	0	1	1	0	1	0	0	0	0	0	0	1	1	1	1	0	0	0
02-658	Human, P	AAG	1	1	0	1	1	0	1	0	0	0	0	0	0	1	1	1	1	0	0	0
17263	Antelope, SA (*3*)	AAG	1	1	0	1	1	0	1	0	0	0	0	0	0	1	1	1	1	0	0	0
17297	Unspecified	AAG	1	1	0	1	1	0	1	0	0	0	0	0	0	1	1	1	1	0	0	0
17783	Antelope, NL	AAG	1	1	0	1	1	0	1	0	0	0	0	0	0	1	1	1	1	0	0	0
17786	Waterbuck, NL	AAG	1	1	0	1	1	0	1	0	0	0	0	0	0	1	1	1	1	0	0	0
17788	Waterbuck, NL	AAG	1	1	0	1	1	0	1	0	0	0	0	0	0	1	1	1	1	0	0	0
17789	Oryx, NL (*3*)	AAG	1	1	0	1	1	0	1	0	0	0	0	0	0	1	1	1	1	0	ND	0
95-114	Waterbuck, NL	AAG	1	1	0	1	1	0	1	0	0	0	0	0	0	1	1	1	1	0	0	0
08-482	Deer, UK	AAG	1	1	0	1	1	0	1	0	0	0	0	0	0	1	1	1	1	0	0	0
10-1561	Cow, BD (*10*)	AAG	1	1	0	1	1	0	1	0	0	0	0	0	0	1	1	1	1	0	0	0
10-1563	Monkey, BD	AAG	1	1	0	1	1	0	1	0	0	0	0	0	0	1	1	1	1	ND	0	0
M. tuberculosis H37Rv	Human, P	AAC	1	1	1	1	1	1	1	1	1	1	1	1	1	1	1	1	1	1	1	1

*RD, region of difference; ND, not determined; LN, lymph node; P, pulmonary tuberculosis; G, gastric fluid; SA, Saudi Arabia; NL, the Netherlands; UK, United Kingdom; BD, Bangladesh. References in parentheses refer to primary publication of the respective strain.
†These isolates tested negative for nitrate reductase and were tolerant to 5 µg/mL thiophene-2-carboxylic hydrazide.

Technical Appendix Table 2. Functional analysis of genes in the RD12 deletion area of *Mycobacterium orygis*

Locus	Gene name
Rv3125c	PPE family protein
Rv3124	Transcriptional regulator
Rv3123	Hypothetical protein
Rv3122	Hypothetical protein
Rv3121	Cytochrome P450 141 (cyp141)
Rv3120	Conserved hypothetical protein
Rv3119	Molybdenum cofactor biosynthesis protein E (moaE1)
Rv3118	Hypothetical protein (sseC1)
Rv3117	Thiosulfate sulfurtransferase (cysA3)
Rv3116	Molybdenum cofactor biosynthesis protein (moeB2)
Rv3115	Transposase
Rv3114	Conserved hypothetical protein
Rv3113	Phosphatase
Rv3112	Molybdenum cofactor biosynthesis protein D (moaD1)
Rv3111	Molybdenum cofactor biosynthesis protein C (moaC1)
